# Oriented Nucleation of both Ge-Fresnoite and Benitoite/BaGe_4_O_9_ during the Surface Crystallisation of Glass Studied by Electron Backscatter Diffraction

**DOI:** 10.1038/srep20125

**Published:** 2016-02-08

**Authors:** Wolfgang Wisniewski, Marek Patschger, Steliana Murdzheva, Christian Thieme, Christian Rüssel

**Affiliations:** 1 Otto-Schott-Institut, Jena University, Fraunhoferstr. 6, 07743 Jena, Germany

## Abstract

Two glasses of the compositions 2 BaO - TiO_2_ - 2.75 GeO_2_ and 2 BaO – TiO_2_ –3.67 GeO_2_ (also known as BTG55) are annealed at temperatures from 680 to 970 °C to induce surface crystallization. The resulting samples are analyzed by X-ray diffraction (XRD) and scanning electron microscopy (SEM) including electron backscatter diffraction (EBSD). Ge-Fresnoite (Ba_2_TiGe_2_O_8_, BTG) is observed at the immediate surface of all samples and oriented nucleation is proven in both compositions. After a very fast kinetic selection, the crystal growth of BTG into the bulk occurs via highly oriented dendrites where the c-axes are oriented perpendicular to the surface. The growth of this oriented layer is finally blocked by dendritc BTG originating from bulk nucleation. The secondary phases BaTiGe_3_O_9_ (benitoite) and BaGe_4_O_9_ are also identified near the surface by XRD and localized by EBSD which additionally indicates orientation preferences for these phases. This behaviour is in contrast with previous reports from the Ba_2_TiSi_2_O_8_ as well as the Sr_2_TiSi_2_O_8_ systems.

Fresnoite in its original form has the composition Ba_2_TiSi_2_O_8_ (BTS). Barium may be partially or completely substituted by Sr to form Sr_2_TiSi_2_O_8_ (STS) while Si may partially or completely be replaced by Ge to form Ba_2_TiGe_2_O_8_ (BTG) or a solid solution. Incommensurate modulations occur in all three compositions^1^. A solid solution between BTG and BTS has also been reported for the entire composition range^2^. While BTS and STS show a tetragonal symmetry, BTG is only tetragonal at high temperatures and shows a displacive phase transition from the tetragonal to the orthorhombic phase^1^ at about 850 °C^3^,^4^. Temperatures of 810, 830 or 870 °C were reported for the phase transition based on differential thermal analysis (DTA)^3^, birefringence measurements^2^,^5^ and X-ray diffraction^5^. Stresses from this transformation are found in single crystals^6^ and crystallographic twins parallel to the (110) or (1–10) planes observed in BTG have been related to it^1^,^4^. These twins may disappear due to heating^3^,^4^ or the application of a uniaxial external stress^3^. In a polycrystalline sample with statistical orientation, the tetragonal phase may be frozen and also occur at room temperature^1^. An additional low temperature phase transition was proposed^3^ and a polar-to-polar phase transition was confirmed at −50 °C (cooling) and 0 °C (heating) in a detailed analysis^4^.

As these crystals are not ferroelectric and thus cannot be poled, the polar BTG crystals must be aligned during crystal growth if piezoelectric properties are required. Apart from growing single crystals in the Czochralski process, glass-ceramic materials containing oriented BTG crystals have been produced via the surface crystallization of glasses^7^,^8^,^9^,^10^,^11^,^12^,^13^,^14^,^15^,^16^,^17^,^18^,^19^,^20^,^21^,^22^,^23^,^24^. Highly oriented glass-ceramics containing e.g. BTS and STS have also been produced using the method of electrochemically induced nucleation (EiN)^25^,^26^,^27^,^28^,^29^. Furthermore, BTG has been crystallized in glasses by laser irradiation^30^,^31^ or drawn into fibers and subsequently crystallized^22^.

Usually, glass compositions with a certain excess of GeO_2_ were chosen to avoid spontaneous crystallization during cooling as well as homogeneous bulk nucleation. In order to suppress bulk nucleation^7^ and to increase the degree of crystal orientation^7^, the surface crystallization experiments have been modified using a temperature gradient^7^,^8^,^9^,^10^,^11^,^12^ or an ultrasonic treatment of the surface^13^. However, other experiments showed that oriented crystallization at the surface also occurs without these experimental efforts^14^,^15^,^16^,^17^,^18^,^19^,^20^,^21^,^22^,^23^,^24^. Applying a strong magnetic field of 10 T during growth has been described to enhance the degree of crystal orientation if applied parallel to the main growth direction and decrease it if the field is applied perpendicular to it^18^. However, the crystal layers contributing to these measurements are only 5–8 μm thick and it is noteworthy that the thickest layer was observed in the sample annealed without a magnetic field^18^. This would allow the conclusion that any magnetic field may reduce the crystal growth rate, especially when applied perpendicular to the growth direction.

The glass forming range in the BaO-TiO_2_-GeO_2_ system has been studied^32^ and the crystallization behavior of a number of glasses was investigated within that vitrification range^23^. It was observed that DTA-measurements in this system are accompanied by a loss of GeO_2_^2^. At least the 22 glass compositions presented in Table 1 have been used to crystallize BTG. While the compositions 1, 10, 12 and 17 were modified by adding SiO_2_, NiO, Fe_2_O_3_, V_2_O_5_, Sb_2_O_3_ or metallic Cu, all other compositions only vary in the concentrations of the basic components BaO, TiO_2_ and GeO_2_. Selected properties of these glasses and the resulting glass ceramics are also stated.

An effect of the glass melting temperature on the optical properties of a glass in this system has been reported^33^,^34^. It was shown that Ti^3+^ is generated in the melt due to the reducing conditions at high melting temperatures^34^ which is in agreement with the well-known observation that redox equilibria are shifted to the reduced state during heating^35^. Hence, the Ti^3+^/Ti^4+^ ratio should increase if the glass is melted at higher temperatures. The presence of Ti^3+^ leads to a decrease in viscosity and network connectivity and gives rise to a dark blue to violet coloration. This has already been shown for the case of fresnoite melts in the BTS system^25^ where increasing the concentrations of Ti^3+^ led to higher nucleation rates and hence promoted crystallization^25^,^36^. This effect has also been quantified in ref. 36. The effect is utilized for the EiN referred to above. Additionally, Pt-droplets were observed in the remelted glass which functioned as nucleation sites during crystallization^33^. The corrosion of Pt was also observed during EiN in the BTS-system where it was concluded that Si^4+^ is reduced to elemental Si which in turn forms a liquid Pt-Si-alloy in the melt. As there are multiple Pt-Ge-alloys^37^ with melting temperatures below 1000 °C, a comparable mechanism may be expected in the GeO_2_ containing glasses considering the reducing conditions at high temperatures.

Crystallized BTG glass-ceramics range from transparent^13^,^15^,^16^,^17^,^18^,^19^,^20^,^21^,^22^,^24^ to opaque^13^,^16^ as an effect of the composition and the crystallization parameters as e.g. also observed in STS containing glass-ceramics^38^,^39^. Polarization micrographs of cross sections have been published^24^ and indicate a strong optical anisotropy. Large second-order optical non-linearities have been found in these materials^13^,^14^,^15^,^16^,^17^,^18^,^19^,^20^,^21^,^22^ and attributed to a preferential orientation of TiO_5_ pyramidal units^13^,^22^. The BTG layer after surface crystallization has been shown to function as a planar optical wave guide^17^. A dramatic increase of the second harmonic intensity in glass-ceramics grown from the composition 2 BaO – TiO_2_ –3.67 GeO_2_ (also known as 30 BaO-15 TiO_2_-55 GeO_2_ or BTG55 in the literature) is observed after annealing at temperatures from 695 to 720 °C while it changes very little from 720 to 740 °C and decreases at 750 °C, probably due to the bulk crystallization triggered by the formation of BaTiGe_3_O_9_^16^. It has been stated that orthorhombic BTG shows a larger optical non-linearity than tetragonal BTG^21^.

While only BTG was detected after crystallizing a stoichiometric melt^17^, phases of the compositions BaTiGe_3_O_9_ (benitoite) and BaGe_4_O_9_ have been reported at the immediate surface after crystallizing glasses containing additional GeO_2_^14^,^16^,^17^. Benitoite was no longer observed in the XRD patterns after removing ca. 2 μm from the sample’s surface via grinding^14^ but it was proposed that it plays a significant role in the nucleation of BTG^16^,^18^,^23^. Based on Raman spectroscopic measurements^16^, it was reported that BTG forms around the initially crystallized benitoite. Other articles suggested that BTG can crystallize without the assistance of the BaTiGe_3_O_9_ phase^18^.

Specific annealing regimes enabled to solely crystallize oriented benitoite from the 2 BaO – TiO_2_ –3.67 GeO_2_ glass at 690 °C^20^,^21^ but BTG is subsequently formed if the annealing time is increased^21^. It was additionally concluded that both tetragonal and orthorhombic BTG occur in this multilayered structure based on XRD-measurements^21^ which is interesting considering that the applied crystallization temperature is below the 830 or 850 °C where BTG shows its high temperature phase transition^1^,^2^,^3^.

XRD-patterns of the surface crystallized layers showed a non-statistical orientation distribution indicating a 001 texture^7^,^9^,^13^,^14^,^16^,^17^,^18^,^19^,^20^,^21^,^22^,^24^ which increased in intensity after 5 μm or more of the surface were removed^9^,^19^,^21^,^24^. Including a nucleation step into the annealing regime was reported to reduce the degree of orientation^9^. Samples annealed at only 695 and 700 °C have been proposed to show XRD-patterns similar to the powder pattern^16^ and it was concluded that the crystalline phase is not oriented at the surface^16^,^17^,^24^. 001 textures have also been indicated by XRD in BTS^40^,^41^ and STS^38^ glass-ceramics, but the application of EBSD showed that the topmost layer shows a different orientation in the case of BTS^40^ which is affected by the annealing temperature^40^. Actually, the analysis of surface crystallized STS from the composition STS+0.45 SiO_2_ provided the first case where the texture detected at the immediate surface also prevailed during growth^39^ while a texture change is observed more often^33^,^40^,^42^,^43^. Hence the assumption of a randomly oriented nucleation in BTG^24^ must be questioned.

A mechanism for the formation of BTG in the 2 BaO – TiO_2_ – 3.67 GeO_2_ glass has been proposed^16^. It was suggested that the presence of differently coordinated Ti^4+^ ions (5 fold instead of 4 or 6) may be related to the crystallization mechanism and that benitoite appears first^16^.

Piezoelectric measurements have shown that the positive end of the poles points towards the growth direction^10^. d_33_-values from 3 to 24 × 10^−12^ m/V have been reported in various glass-ceramics^7^,^8^,^9^,^10^,^11^,^12^,^13^,^14^,^15^ but it is noteworthy that Takahashi *et al.* used the Marker Fringe technique and different refractive indices in the calculations for some articles^13^,^14^. The d_33_-values increase if the topmost layer of crystals is removed and are hence linked to the degree of crystal orientation in the glass-ceramic^19^. The values obtained using the Marker Fringe technique dramatically exceed the d_33_-value of 10 × 10^−12^ m/V stated for BTG single crystals^7^,^9^ using a d_33_-meter.

Due to the properties outlined above, fresnoite based glass ceramics have been proposed for applications as hydrophones^11^, piezoelectric components or active photonic glass fibers^22^.

The application of electron backscatter diffraction (EBSD) to the surface crystallization of glass-ceramics containing BTS^40^,^41^ and STS^38^,^39^,^42^ has significantly altered the understanding of occurring crystal orientations and textures in these systems. For example, the observation of oriented nucleation in these glass-ceramics^38^,^39^,^40^,^41^,^42^, also observed e.g. during the crystallization of a BaO·Al_2_O_3_·B_2_O_3_ glass^43^, challenges the basic assumption of oriented nucleation in the classic nucleation theory for glasses. It must not be confused with the oriented growth of crystals in glasses which has been known for many years.

However, EBSD has not yet been applied to BTG. A short introduction concerning the method and its application to fresnoit glass-ceramics is found in ref. 40. In this article, we apply EBSD to two glasses: glass 1 is of the composition 2BaO · TiO_2_ · 2.75GeO_2_ were 0.75 mol% of additional GeO_2_ are added to the stoichiometric BTG composition to enable the direct comparison to previous analysis featuring BTS^40^,^41^ and STS^38^,^42^ glass-ceramics. This composition is relevant because its BTS-equivalent has been used to grow piezoelectric, macroscopic single crystals with a cross section of more than 1 cm^2^ and 10 cm long^28^. Glass 2 of the composition 2 BaO – TiO_2_ –3.67 GeO_2_, has been intensively studied for its optical non-linearity (see Table 1) and a dual layered surface crystallization has been described in this system^22^.

## Results and Discussion

### Glass 1: the composition 2BaO - TiO_2_ - 2.75 GeO_2_

**Figure 1:**
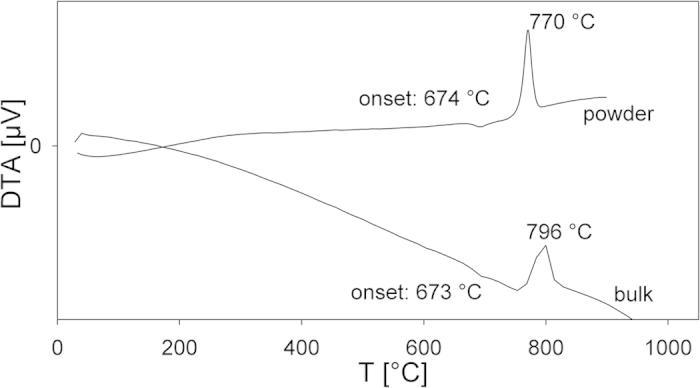
DTA measurements of the bulk and powdered 2BaO · TiO_2_ · 2.75GeO_2_ glass. The error in the temperatures evaluated from the DTA profiles is ±3 °C.

The obtained glass was slightly yellow and transparent. As the extinction coefficients of Ti^3+^ are very high and a violet coloration is not observed, it is unlikely that there is enough Ti^3+^ in the glass to show a measurable effect on the nucleation and crystallization behavior. The DTA profiles measured from a compact and a powdered sample of the current glass are presented in Fig. 1. The glass transition temperature T_g_ was determined to be 673 and 674 °C while exothermic peaks, the crystallization peak T_x,_ occurred at 770 and 796 °C respectively.

**Figure 2:**
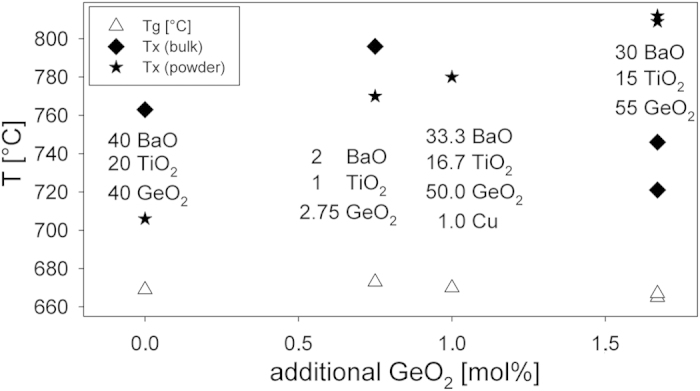
T_g_ and T_x_ values of the glass compositions in Table 1 only modified in the amount of added GeO_2_ as well as the composition 1 Cu-33.3 BaO-16.7 TiO_2_-50 GeO_2_.

Figure 2 compares the T_g_ and T_x_ values of the glass in this article with those stated in the literature concerning the other glass compositions from Table 1 only modified in their GeO_2_ concentration. The composition containing a small amount of Cu is included because the effect of Cu on the T_g_ is deemed minimal. While T_g_ remains relatively constant, T_x(powder)_ increases with the increasing GeO_2_ concentration. In contrast T_x(bulk)_ seems to have a maximum somewhere between adding 0.75 and 1.66 mol% of GeO_2_.

The phases benitoite (BaTiGe_3_O_9_) and BaGe_4_O_9_ are expected to only form locally and close to the surface of the BTG glass-ceramics which poses an ambiguity problem for building the material files necessary for EBSD analysis. To improve the reliability of these material files, powders of both phases were produced via solid-state reactions. Material files were then built and optimized using the EBSD-patterns obtained from the respective powder and the Inorganic Crystal Structure Database (ICSD) files 36185 and 83734.

ICSD-file 36185 features hexagonal benitoite of the composition BaTiSi_3_O_9_, i.e. the Si-equivalent of Ge-benitoite expected in the glass-ceramics produced here. Using this data is judged to be acceptable because the indexing procedure of EBSD mainly focuses on the crystallography of the phase and none of the databases accessible to us (ICSD, AMCSD, COD) currently contain an entry with the Wycoff-positions of Ge-benitoite which are, however, necessary for building a material file in the given software. As a rule of thumb, a pattern may be considered reliably indexed in the TSL software if the primary orientation solution receives at least 30 votes, a fit of less than 1° and a Confidence Index (CI) value larger than 0.100.

The material file of hexagonal benitoite performed very well for single patterns as well as in a scan of the embedded benitoite powder. In the case of trigonal BaGe_4_O_9_, the material file also showed very good results but a confidence index of 0.000 for almost all detected orientations due to pseudo symmetries where the first and second orientation solutions receive the same number of votes. This problem also occurred during the analysis of surface crystallizing BaAl_2_B_2_O_7_ glass-ceramics^43^. A material file based on ICSD file No. 15674 (BaGe_4_O_9_ but with space group 143 instead of 150) led to practically identical results. Thus the CI fails as a reliability indicator in its usual form in the case of the material files built for BaGe_4_O_9_. If, however, an orientation solution receives enough votes and a sufficient fit factor in combination with a CI of exactly 0.000, this may be considered as a correct orientation but with an ambiguity of a 180° rotation around the [0001] direction.

The real problem for the analysis of benitoite and BaGe_4_O_9_ is that the achieved material files may be used to index the EBSD-patterns of both phases, making a phase separation based on EBSD alone unreliable. This type of problem may be solved by either fine tuning the material files based on characteristic kikuchi bands, e.g. for the separation of cubic and hexagonal ZnS^44^, or by chemistry assisted indexing (ChI) which combines EBSD with energy dispersive X-ray spectroscopy (EDS) to enable a chemistry based phase attribution to a data point, e.g. used to separate the cubic phases YAG and yttrium stabilized zirconia^45^. The first approach has been unsuccessful so far and ChI is unsuitable for the given problem because both phases contain Ba and the only suitable separator of the phases would be via the Ti content. Reliably separating Ba and Ti in the given SEM, however, is only possible using the enhanced resolution of wavelength dispersive X-ray spectroscopy (WDS) instead of EDS, and WDS cannot be performed with the tilted SEM-stage necessary for EBSD.

Two entries concerning the low temperature phase of BTG can be found in the ICSD data base: file no. 39133 (a = 12.310 Å, b = 12.292 Å, c = 5.366 Å) and file no. 281271 (a = 12.301 Å, b = 12.284 Å, c = 10.737 Å). While the a and b axes almost have the same values in both files, the c-axis in file 281271 is basically twice as long as in file 39133 because it was doubled in order to describe a modulated structure at room temperature^6^. The main challenge for the EBSD analysis of BTG, however, should be the minimal difference between the a and b axes of only ~0.017 Å, i.e. ~0.14%. Some work concerning a similar problem with cubic zirconia compared to tetragonal zirconia, which has only a slight tetragonal distortion, has been presented by Martin *et al.*^46^.

**Figure 3:**
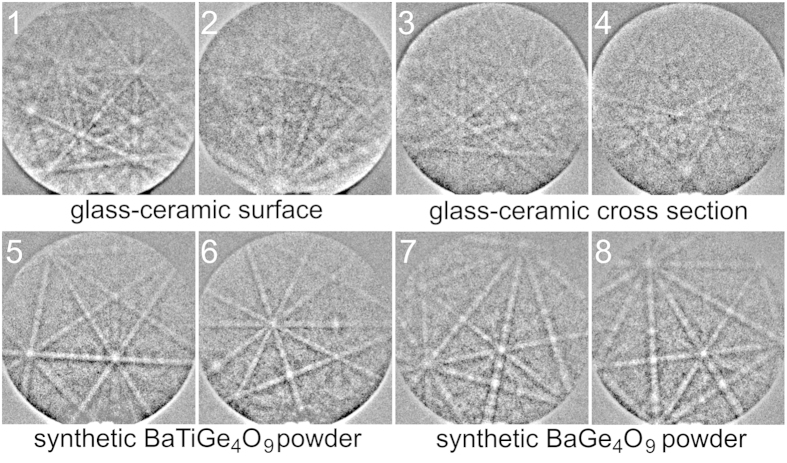
EBSD-patterns acquired from the untreated surface (1,2) and polished cross section (3,4) of the BTG glass-ceramics as well as from synthesized powders of benitoite/BaTiGe_3_O_9_ (5,6) and BaGe_4_O_9_ (7,8).

The EBSD-patterns presented in Fig. 3 were indexed using parameters comparable to those applied during EBSD-scans later on to test the applicability of the material files, selected indexing parameters are stated in Table 2. The patterns 1 and 2 were obtained directly from the crystallized surface of a BTG glass-ceramic while the patterns 3 and 4 were obtained from a polished cross section. The patterns 5 and 6 originate from the benitoite powder while the patterns 7 and 8 originate from the BaGe_4_O_9_ powder.

The patterns 1–4 are clearly indexed as BTG using a material file based on the ICSD file 39133. Despite the minimal difference between the a- and b-axes in the material file, the indexing parameters indicate a very reliable orientation solution and a clear separation between BTG and benitoite/BaGe_4_O_9_ is achieved. Additionally, the position of the 001-pole is constant for the first two orientation solutions for these patterns. Hence even if the CI-value drops below 0.1 there is still a good chance that the orientation of the c-axis is correct.

Apart from some fit values slightly larger than 1°, the patterns 5–8 receive reliable indexing parameters for both benitoite and BaGe_4_O_9_. These fit values can easily be decreased by choosing a smaller interplanar angle tolerance (IAT) than the 3° used here which, however, proved to be less useful during the EBSD-scans. The CI-values of the BaGe_4_O_9_ file are always 0.000 due to the pseudo symmetry problem outlined above, making the CI-value useless as a phase differentiating factor in this case. The patterns 6 and 8 receive exactly the same number of votes from both material files and pattern 5 receives almost the same fit factor. Activating the band width ratio matching function did not lead to a satisfactory phase separation either.

These results show that the orientation measurements are satisfactory and BTG may be reliably separated from benitoite/BaGe_4_O_9_. The latter two phases may not be separated reliably at this point and hence only the material file of benitoite is used for further measurements as it does not suffer from the pseudo symmetry problem of the BaGe_4_O_9_ file. The three phase system in the glass-ceramic is thus reduced to the two phase system of BTG and benitoite/BaGe_4_O_9_ for further analysis.

As it turned out in the subsequent measurements, the grain CI-standardization clean-up procedure, which only changes CI-values but no crystal orientations, provided sufficient datasets for analysis while still using a CI-filter to remove unreliable data points originating e.g. from the residual glass.

**Figure 4:**
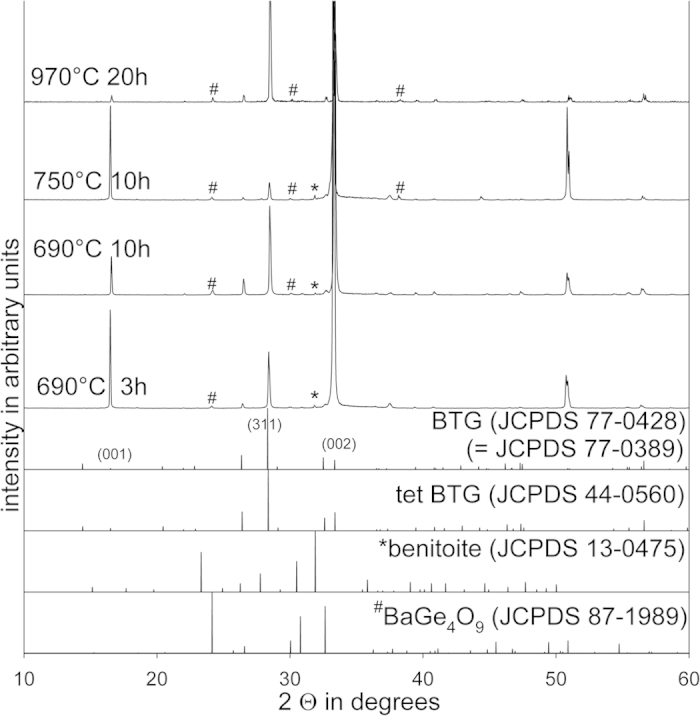
XRD patterns obtained from the untreated surfaces of compact samples of glass 1. The theoretical patterns of tetragonal and rhombohedral BTG, benitoite as well as BaGe_4_O_9_ are presented for comparison.

Polished samples of the glass 1 were annealed at temperatures from 690 to 970 °C in order to achieve comparability with the surface crystallization research performed on glass-ceramics containing BTG^7^,^8^,^9^,^10^,^11^,^12^,^13^,^14^,^15^,^16^,^17^,^18^,^19^,^20^,^21^,^22^,^23^,^24^ as well as BTS^40^,^41^ and STS^32^,^39^. Considering the phase transition of BTG at around 850 °C^34^ (810–870 °C^2^,^3^,^5^), the question arises if there are any significant differences in the crystallization of BTG grown at temperatures below 810 °C or above 870 °C. In both cases, nucleation should occur in the orthorhombic modification. XRD-measurements were performed on the direct surfaces of compact samples and the resulting patterns in Fig. 4 show an exaggeration of the (00n) peaks of BTG which is in agreement with the literature^7^,^8^,^9^,^10^,^11^,^12^,^13^,^14^,^15^,^16^,^17^,^18^,^19^,^20^,^21^,^22^,^24^ and indicates an 001 texture of surface crystallized BTG within the information depth of XRD. Additional peaks not attributable to BTG occur in all patterns, the peak at ~31° may be attributed to benitoite while the peaks at ~24°, ~30° and ~38° are attributable to BaGe_4_O_9_. While these weak peaks are not a clear proof that these phases, also described for other BTG containing glass ceramics^14^,^16^,^17^, occur in these samples, their presence in small amounts seems very probable.

**Figure 5:**
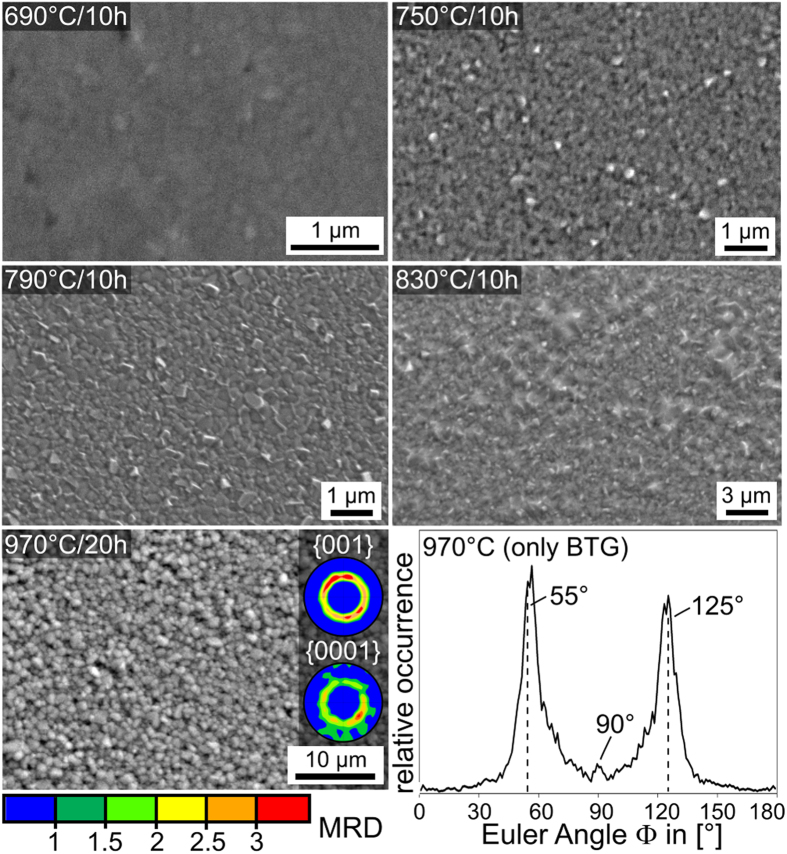
SEM-micrographs of the sample surfaces after annealing glass 1 at the stated temperatures. The {001} and {0001} PF of textures calculated from the BTG and benitoite datasets of an EBSD-scan performed on the sample annealed at 970 °C are presented as well as the distribution of the Euler Angle Φ in the BTG-dataset of the scan.

SEM-micrographs obtained from the surfaces of samples annealed at the stated temperatures are presented in Fig. 5. The small crystal sizes of less than 1 μm indicate a very high nucleation rate at the surface of this glass. While single EBSD-patterns reliably indexable as BTG could be acquired locally from all these surfaces, only the sample annealed at 970 °C provided EBSD-patterns from a representative surface coverage to enable texture analysis. A scan of 247150 data points was performed with a step size of 200 nm on this surface where 71% of the data points received a CI>0.1 after applying the grain CI-standardization. Of these reliably indexed data points, 82.4% were attributed to BTG and 17.6% to benitoite/BaGe_4_O_9_ which cannot be reliably separated at this time as outlined above. Hence a complete layer of benitoite comparable to that proposed to occur at the surface of the glass BTG55 crystallized at 690 °C^20^ is impossible in this sample. The 29% of data points excluded from the analysis can be attributed to an insufficient pattern quality due to grain boundaries or residual glass. The {001} and {0001} PFs of textures calculated from the BTG and benitoite data sets in this surface scan are superimposed on the micrograph and the distribution of the Euler Angle Φ for BTG in the scan is presented. Φ describes the tilt of the c-axis of BTG in this system and a value of 90° indicates a c-axis oriented perpendicular to the sample surface. The ring in the {001} PF was reproduced in 5 of 5 scans performed on two separately prepared samples while a weak, central dot only occurred in 3 of the 5 scans. However, a small peak at 90° is observed in the Φ-distribution. Hence the 001 texture indicated by this central probability maximum is very weak compared to the texture indicated by the ring. Due to the very limited information depth of EBSD, which is still a matter of debate^47^, the ring in the PF of BTG and the peaks at Φ = 55, 125 and 90° indicate that the topmost crystals of BTG already show a non statistical orientation distribution in the form of two textures: one with the c-axes preferably tilted by 35 ± 10° from the surface normal and the other with the c-axes parallel to the surface normal. A similar ring is observed in the {0001} PF based on the data attributed to benitoite/BaGe_4_O_9_. As both phases occur according to the XRD-measurements in Fig. 4 but could not be separated in the EBSD-dataset, it is impossible to state whether this texture originates from benitoite, BaGe_4_O_9_ or both at this point.

A similar ring texture indicating a c-axis tilt of 58° at the surface was detected in the BTS equivalent of the current glass composition after crystallization at temperatures from 790 to 970 °C^40^,^41^ while the c-axis was perpendicular to the surface in the STS equivalent at 970 °C^38^. Although the (101)-plane of the BTS unit cell is perpendicular to the surface if the c-axis is tilted by 58°, this is not the case for the BTG unit cell, independent of whether a c-axis value of 5.366 Å or 10.737 Å is assumed. Hence the [101] direction is not the rotation axis of the orientation preferred during the nucleation of BTG.

**Figure 6:**
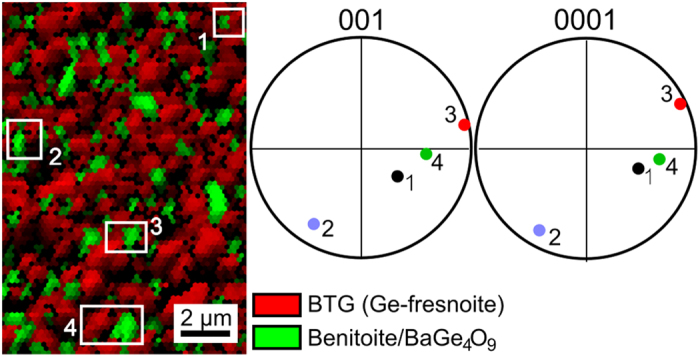
Phase+IQ-map of an EBSD-scan performed on the surface of a glass 1 sample annealed at 970 °C for 20 h. The 001- and 0001-poles of the areas attributed to BTG and benitoite/BaGe_4_O_9_ in the frames 1–4 are presented.

The common texture observed in Fig. 5 raises the question whether there is a crystallographic relationship between BTG and benitoite/BaGe_4_O_9_. Figure 6 shows the combined phase+image quality (IQ)-map of an EBSD-scan performed on the same surface where the textures were measured. The frames 1–4 highlight areas where the 001-poles of BTG are positioned very close to the 0001-poles of the patterns indexed as benitoite. While these poles share a similar position in the respective PFs, this observation is not valid for most of the grains and a true epitaxial relationship is not detected. Due to the outlined indexing problem a statement whether this relationship occurs in benitoite, BaGe_4_O_9_ or both cannot be made at this point. Due to the small grain size and the topography of the surface a validation of the phases using WDS is deemed unreliable. Larger crystals of the non-BTG phases could help solve this problem in the future.

**Figure 7:**
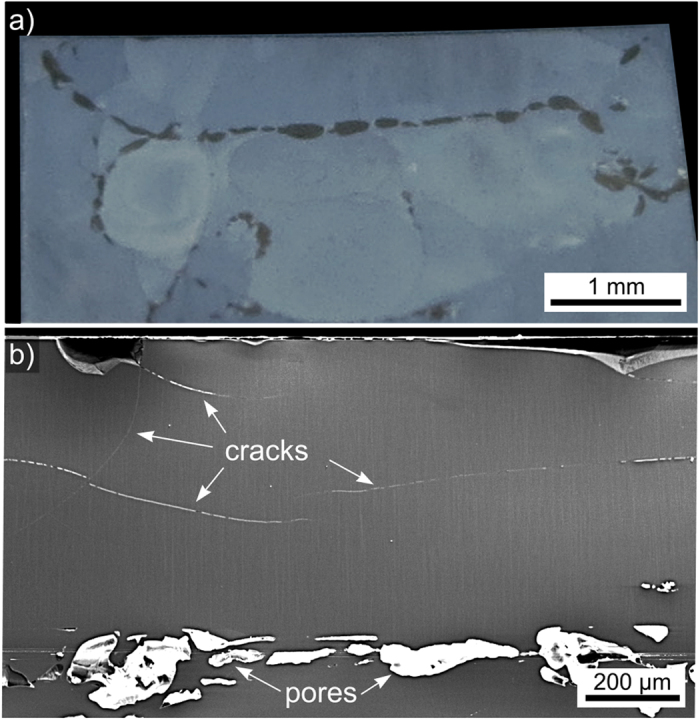
(**a**) Photograph of the cross section of a glass 1 sample annealed at 790 °C for 10 h. (**b**) SEM-micrograph of a similar cross section from a glass 1 sample annealed at 750 °C for 10 h.

**Figure 8:**
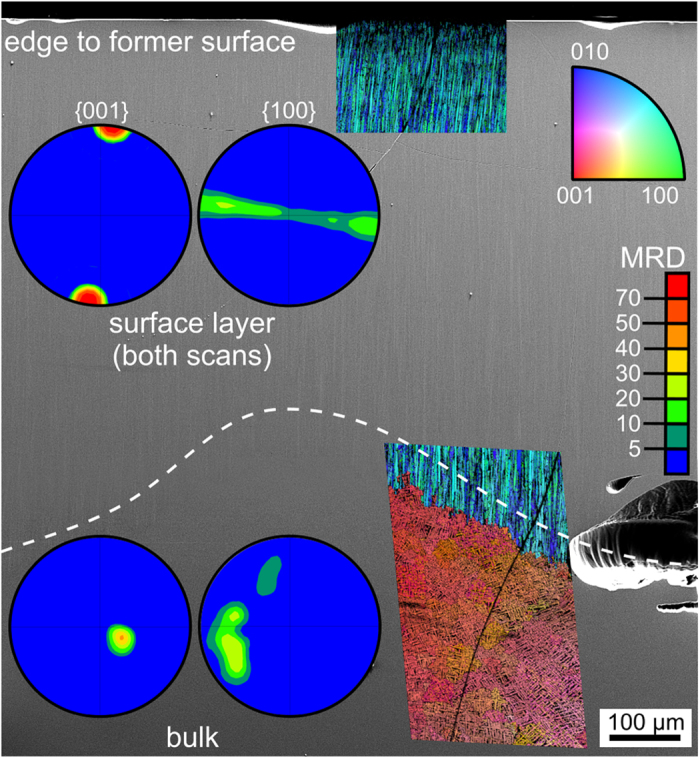
SEM-micrograph of the cross section of a glass 1 sample annealed at 750 °C for 10 h. IPF+IQ-maps of EBSD-scans performed on the area as well as 001 and 100 PFs of textures calculated from the surface layer and the bulk are also presented.

Cross sections of these samples were prepared in order to analyze the crystal growth into the bulk. Figure 7a) shows the cross section of an opaque sample annealed for 10 h at 790 °C. Large pores systematically occur where crystal growth fronts collided, i.e. between the oriented layers growing from the sides as well as between them and the bulk crystallization. Additionally, some far ranging cracks are observed in Fig. 7b). Due to the position and shape of the cracks and pores it seems plausible that they relax stresses caused by the density increase during crystallization. A cross section SEM-micrograph of the surface crystallized layer in a sample annealed at 750 °C for 10 h is featured in Fig. 8. The micrograph is superimposed by the combined inverse pole figure + IQ maps (IPF+IQ-maps) of EBSD-scans performed at the edge to the former surface (top) and at the interface to the bulk crystallization. The latter is illustrated by the dotted line, not plane parallel to the surface and pores frequently occur here as already illustrated by Fig. 7. Please note that the crystallization front is plane-parallel to the surface in samples with a thin layer of crystals, both in this glass composition and in others presented in the literature^13^,^14^,^16^,^17^,^18^,^22^,^24^. {001} and {100} PFs of textures calculated from the BTG-dataset of both crystallization zones are presented. An area of growth selection similar to that observed within the first ~10 μm of growth in the BTS-glass of the same molar composition^40^ was not detected in any sample. Some growth structures at a certain distance from the surface show diameters of more than 5 μm, i.e. they are much larger than the crystals observed at the immediate surface, see Fig. 5. Even cross sections of samples with a crystal layer of only 10 μm showed a strong 001 texture, allowing the conclusion that the kinetic selection from the texture at the surface to the 001 texture during growth into the bulk happens extremely fast in this system, probably due to the very high nucleation rate. In the STS system, the speed of the kinetic selection during growth into the bulk notably increased if the nucleation rate was increased via surface modifications^42^. Although the texture of the bulk crystallization indicates that the c-axes of the entire area are almost perpendicular to the surface crystallization, the degree of orientation is significantly reduced. Additionally, the {100} PF from the surface crystallized layer indicates a random rotation of the crystals around the c-axes while the {100} PF from the bulk does not. A scan performed on 430 × 700 μm^2^ of the bulk crystallization in a sample annealed at 790 °C for 10 h showed multiple orientations over larger areas, i.e. bulk nucleation occurs in this system but with a low nucleation rate.

Only 1.1% of the reliably indexed data points in the surface near scan are attributed to benitoite/BaGe_4_O_9_. The non-BTG areas basically occur parallel to the BTG growth structures, are less than 1 μm thick but frequently reach several μm in length. Assuming they are composed of benitoite the c-axes would be generally oriented parallel to the c-axes of the neighbouring BTG but with a lower degree of orientation and without an epitaxial relationship. Some data points attributed to benitoite/BaGe_4_O_9_ are observed more than 100 μm below the surface but they do not occur at all in scans covering the boundary between surface and bulk crystallization. Hence it appears that benitoite/BaGe_4_O_9_ crystallizes in minimal amounts during the initial growth of the oriented surface layer whenever the local stoichiometry is suitable.

**Figure 9:**
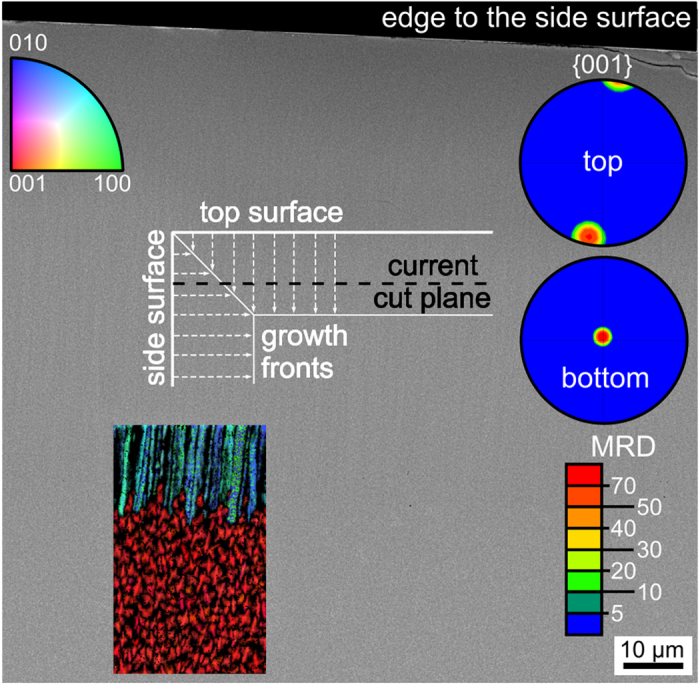
Cut plane parallel to surface, but ca. 50 μm below the surface. Contact to the surface crystallized layer from the side. The current cut plane is schematically outlined. Only BTG is included in the scan.

Due to the minimal amounts of benitoite/BaGe_4_O_9_ observed below the surface, the following measurements only feature results concerning BTG. To confirm the observed bulk nucleation and gain further insight into the microstructure of BTG in the oriented layer, part of the sample featured in Fig. 8 was cut parallel to the initial surface. Figure 9 presents this cut plane, outlined in the figure, approximately 50 μm below the initial surface, at the interface between the oriented crystalline layers resulting from surface nucleation from two sides which are perpendicular to each other. The {001} PFs of textures calculated from the top and bottom parts of the scan show that the BTG-crystals in both areas are oriented perpendicular to each other and show the extreme degree of c-axis alignment also observed in the surface layer featured in Fig. 8.

**Figure 10:**
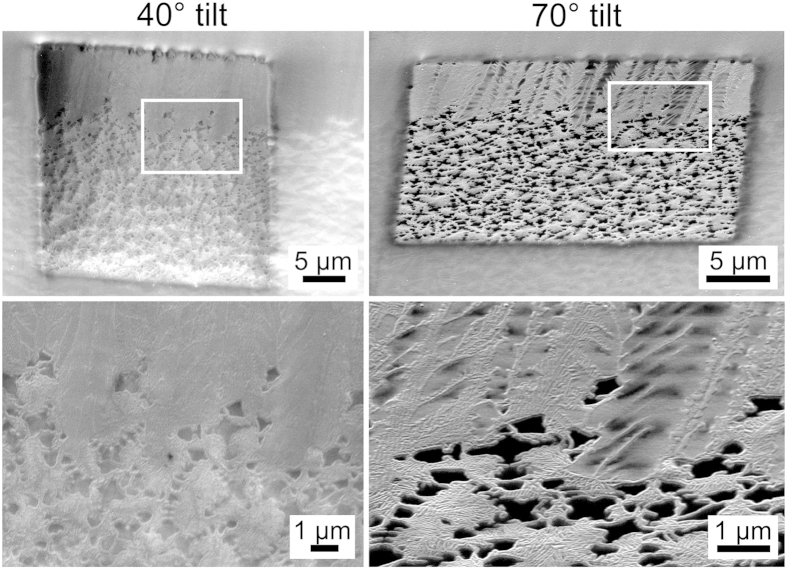
Insight into the BTG microstructure through enhanced topography contrast after an EBSD-scan.

While the contrast in the SEM-micrograph is very low, the IPF+IQ-map of the performed EBSD-scan shows that the BTG growth structures appear as lines when viewed from the side but as stars when viewed parallel to their growth direction. As the EBSD-scan was performed with a step size of 200 nm, the thermal footprints of the scan step overlap which can lead to increased temperatures in the scan area and even cause EBSD-pattern degradation^48^. In the case of this scan, a significant increase of the topography contrast was observed after the scan, probably because some of the residual glass at the surface was evaporated. Thus details of the growth structure morphologies could be visualized by tilting the scanned surface. SEM-micrographs of the growth structures in the scanned area are presented in Fig. 10 where the framed areas are presented in greater detail below the overview images. The BTG-crystals cut parallel to their c-axes clearly show dendritic morphologies. The dark areas between the BTG-crystals cut perpendicular to their c-axes did not provide any EBSD-patterns and show the location of the residual glass. It may be concluded that the oriented layer of surface crystallization is composed of aligned BTG dendrites which are almost limited to their primary core and show few secondary or ternary structures due to the limited space available for growth.

**Figure 11:**
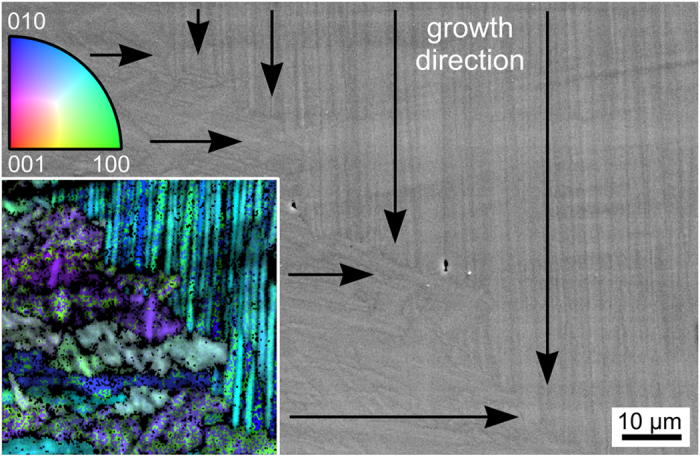
SEM-micrograph and IPF+IQ-map of growth front interaction.

Figure 11 presents an SEM-micrograph of the area where the growth front from the initial surface collides with a growth front originating from the side of the sample. The IPF+IQ-map of an EBSD-scan performed on a similar area is also presented. The steps observed at the interface show that these fronts simply block each other upon collision and the growth velocity is neither accelerated nor depressed. This is in contrast to the growth front interaction in the STS-equivalent of the studied BTG glass where the growth velocity is increased by the growth front interaction^32^.

The method of EBSD-pattern degradation was applied to polished and unpolished surfaces using the same conditions stated in ref. 48. In contrast to comparable measurements performed on BTS^48^ and STS^32^ using a step size of 10 nm, clear problems already occurred during the measurements on these BTG glass-ceramics using a step size of 100 nm under the same experimental conditions. However, the microstructure of these glass-ceramics basically makes it impossible to perform these measurements on an area completely composed of crystalline material. Hence, it cannot be clearly deduced whether the BTG crystal is much more sensitive to the electron radiation, it has the lowest melting point of the three phases, or whether the sensitivity is caused by the remaining glassy matrix. A statement concerning a glass skin covering the crystals is hence also not possible.

These results are observed in all samples annealed at temperatures in the range from 750 to 970 °C for 10 to 20 h. Obvious differences between the crystallization below and above the phase transition temperature of BTG were not observed except for a decrease of the nucleation rate and the fact that higher annealing temperatures tend to enable a better EBSD-pattern acquisition from the untreated surface. Perhaps the long annealing times at high temperatures allow some of the twinning probably present in the system^3^,^4^ to dissipate. Both a larger crystal size and a lower number of twins would enhance the ability to achieve EBSD-patterns. In samples annealed at 690 °C, the crystal layers were ca. 4 μm thick after 3 h and less than 20 μm thick after 10 h while bulk nucleation was not yet detected. Due to the preparation problems concerning the top 10 μm of crystallization and the sensitivity of these materials towards the electron beam, a detailed analysis of these thin crystal layers could not be achieved at this point. The EBSD-measurements that were possible indicate a 001 texture of BTG in these layers which is in agreement with the XRD-result of Fig. 4 and the literature.

### Glass 2: the composition 2 BaO – TiO_2_ –3.67 GeO_2_

The prepared glass was transparent and showed the same yellow coloration already observed in glass 1. A Differential scanning calorimetry (DSC)-measurement of the glass showed the glass transition temperature T_g_ to be 674 °C and the onset of crystallization occurred at ca. 757°C. Samples were annealed at temperatures from 680 to 970 °C for times from 0.5–12 h and in a two-step process at 690 °C for 3 h with another 3 h at 720 °C in analogy to ref. 21.

**Figure 12:**
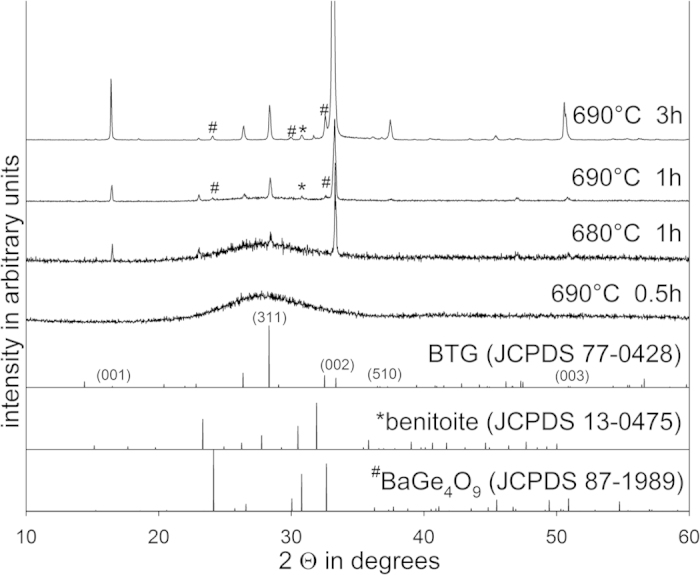
XRD-patterns obtained from compact samples of the glass 2 annealed with the stated regimes. The theoretical patterns of rhombohedral BTG, benitoite as well as BaGe_4_O_9_ are presented for comparison.

The XRD-patterns presented in Fig. 12 obtained from some of these samples show that the pure layer of BaTiGe_3_O_9_ (benitoite) observed in ref. 21 could not be achieved, even by reducing the annealing time to only 1 h and lowering the annealing temperature to 680 °C. Instead, the pattern obtained from the sample annealed at 680 °C only shows peaks attributable to BTG while the patterns of the samples annealed at 690 °C for 1 and 3 h respectively additionally indicate benitoite and BaGe_4_O_9_. Annealing for only 0.5 h at 690 °C did not provide any indication of crystallization in the XRD patterns and a subsequent analysis of the sample surface using SEM/EBSD also failed to produce any indication of crystallization.

**Figure 13:**
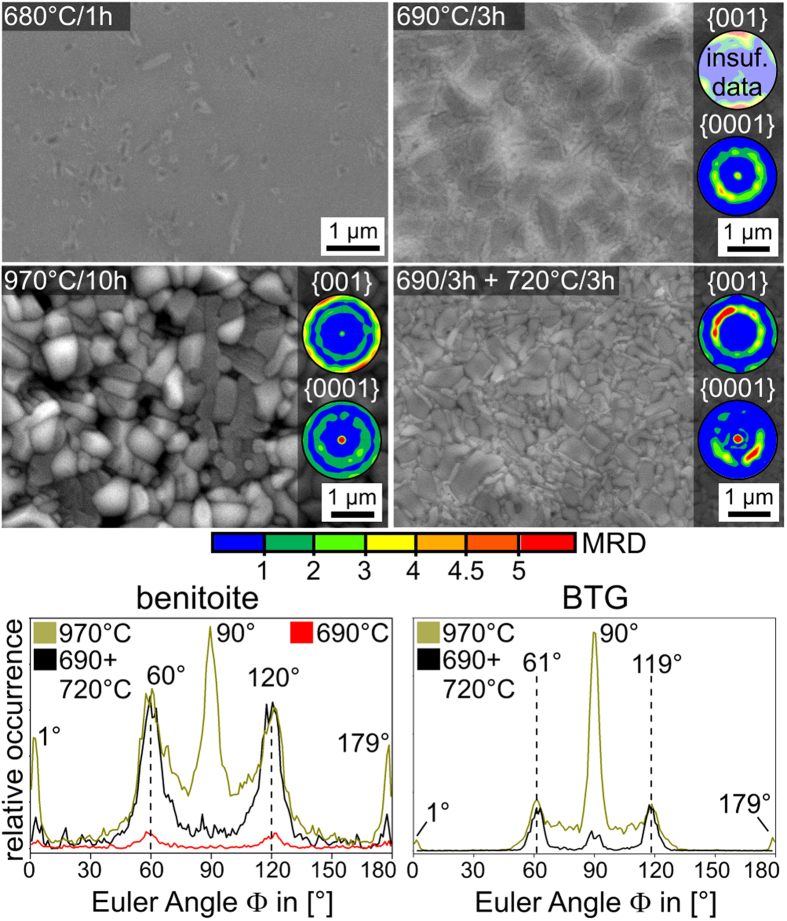
SEM-micrographs featuring the surfaces of glass2 samples annealed using the sated regimes. {001} and {0001} PFs of textures calculated form filtered data sets of EBSD-scans performed on these surfaces are presented.

Figure 13 shows the surface microstructures of glass 2 samples annealed using the stated regimes. Both individual crystals and uncrystallised glass are observed after 1 h at 680 °C and the corresponding XRD-pattern in Fig. 12 already indicates a 001 texture via the exaggerated (00n) peaks although a significant growth selection cannot have occurred in this sample. EBSD-measurements could not be performed on this surface because the crystals failed to provide EBSD-patterns of sufficient quality. All other surfaces are fully covered by crystals slightly larger than those featured in Fig. 5, show a clear topography and contain at least two phases which is in agreement with the XRD-results presented in Fig. 12. WDS-measurements were attempted but the up to 1 μm large crystals are still too small for a reliable phase localization given the high current necessary for the analysis.

EBSD-patterns reliably indexable as either BTG or benitoite/BaGe_4_O_9_ were obtained from all surfaces. Despite the strong topography, EBSD-scans were performed so that {001} and {0001} textures could be calculated. Relevant information concerning the datasets used for these calculations are stated in Table 3. While two of the scans are based on an unrepresentative percentage of the scan data (7.2% and 22.4%), the number of reliable data points is sufficient for texture calculations except for the PF of BTG in the sample annealed at 690 °C for 3 h. The texture data of the sample annealed at 970 °C for 10 h is, however, based on 75.8% of the scan data and hence deemed representative. As all orientations occur in these scans, the possibility of an orientation specific EBSD-pattern acquisition may be excluded, allowing the conclusion that the low indexing rate is mainly due to the topography and the small crystal size. Increasing annealing temperatures led to an increased percentage of BTG in the data sets from only 3.3% after crystallizing at 690 °C for 3 h to 34.2% after crystallizing at 970 °C for 10 h.

In analogy to glass 1, annealing at higher temperatures increases the indexing rate significantly as well as the detected amount of BTG at the immediate surface. While the double texture observed in glass 1 occurs in almost all calculated textures, the phases in the sample annealed at 970 °C for 10 h show an additional orientation preference for crystals with their c-axes oriented parallel to the surface. This preference is also observed in the texture of BTG obtained from the sample crystallized via the two step process. As with glass 1, it is impossible to state whether these textures originate from benitoite, BaGe_4_O_9_ or both at this point. The Euler angle distributions presented below show that the c-axes of BTG are now predominantly tilted by 29° from the surface normal in contrast to the 35° observed for glass 1 in Fig. 5. Again, the textures of BTG and benitoite/BaGe_4_O_9_ are basically the same.

**Figure 14:**
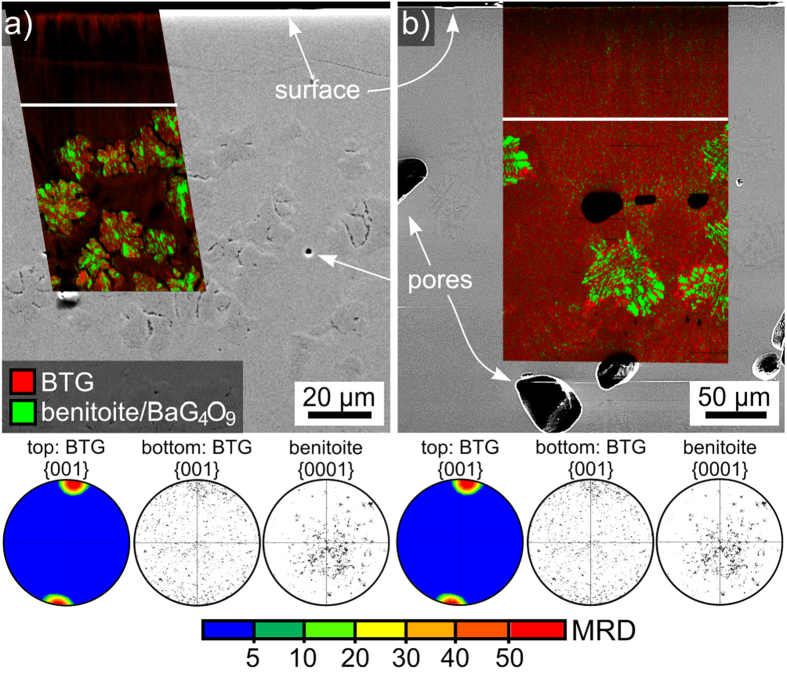
SEM-micrographs of cross sections prepared from samples of glass 2 annealed (**a**) at 690 °C for 12 h and (**b**) at 970 °C for 10 h. Phase+IQ-maps of EBSD-scans performed on the respective areas are superimposed on the micrographs. PFs of textures calculated for BTG from filtered data sets of much larger scans are presented for the top layer of surface crystallization while PFs of both BTG and benitoite are presented for the bottom parts of the scans representing the bulk crystallization.

Cross sections of various samples were prepared and analysed. Figure 14a presents the SEM-micrograph of a cross section of a sample annealed at 690 °C for 12 h while Fig. 14b presents equivalent data for a sample annealed at 970 °C for 10 h. In both cases, the top layer of crystallization appears homogeneous but heterogeneous growth structures are observed in the bulk after a) ca. 30 μm and b) ca. 100 μm. The superimposed phase+IQ-maps of performed EBSD-scans show that only minimal amounts of benitoite/BaGe_4_O_9_ are detected by EBSD in the top layer while the bulk structures are composed of BTG as well as benitoite/BaGe_4_O_9_. Texture information of more representative scans covering a) 300 × 300 μm^2^ and b) 260 × 100 μm^2^ are presented to show that the top layer of mainly BTG is highly oriented with the c-axis perpendicular to the surface while no clear texture is observed in the bulk for either phase. Sample preparation is very challenging in these samples because the crystals are much finer than those observed in glass 1. Instead of the systematic pores at the growth front observed in Fig. 7 these samples contain irregularly spaced pores throughout the bulk. These results indicate that glass 2 shows a bulk nucleation much closer to the surface than glass 1, making glass 1 more suited for achieving thick layers of 001-oriented BTG.

The results presented above indicate that BTG and secondary phases such as benitoite and BaGe_4_O_9_ occur at the surface of almost all samples. Only crystallizing glass 2 at 680 °C for 1 h provided a one phase system of BTG according to the XRD-pattern, which is not very well suited to exclude the existence of a phase, instead of the benitoite detected in ref. 21 using a comparable annealing regime. A confirmation by EBSD was not possible in this case due to insufficient EBSD-pattern quality. Additionally, the XRD-measurement of this sample showed enhanced (00n) peaks while the SEM-micrograph of its surface showed an incompletely crystallized surface, basically excluding growth selection as an orientation mechanism. Hence the 001 texture indicated by XRD in this sample may also be seen as confirmation of the oriented nucleation of BTG.

A complete layer of either phase at the surface is impossible in all other samples as EBSD-patterns reliably indexable as BTG were obtained from the surface of every annealed sample which is in agreement with ref. 18. The fact that both BTG and benitoite have formed first in glasses which should have the same composition indicates that e.g. the production process or varying impurities in the raw materials may significantly affect the order of nucleation in this system. While the two-step growth model outlined in ref. 21 cannot be valid for the experiments presented above, a predominant occurrence of benitoite/BaGe_4_O_9_ at 690 °C was observed by both groups, e.g. only 3.3% of the data points of the corresponding EBSD-scan in Fig. 13 are attributed to BTG. Increasing the annealing temperature leads to an enhanced detection of BTG at the immediate surface, i.e. 34.2% after crystallizing at 970 °C for 10 h. It must be noted, however, that the crystal size may have an effect on the EBSD-pattern formation. If for example the BTG crystals are in the sub-μm range while the crystals indexed as benitoite are in the μm-range, this would imply a lower surface fraction of BTG than actually occurs.

Due to the high nucleation rate, the high fraction of BTG in samples crystallized at 970 °C in both glasses and the sole occurrence of BTG in glass 2 at 680 °C, it seems acceptable to assume that BTG nucleated independently from the other phases in these experiments. This means that oriented nucleation is indicated for BTG as well as for at least one of the secondary phases. As oriented nucleation was also observed after the crystallization of two glasses in the STS system^32^,^39^ and over a wide temperature range of BTS surface crystallization in a comparable glass composition^41^, the assumption of a randomly oriented nucleation in ref. 24 and more generally in the classic nucleation theory for glasses must be questioned.

A temperature dependent texture formation similar to that observed in the BTS system^41^ is indicated in glass 2 which proved to be more suited for EBSD-analysis of the surface. In contrast, the bulk crystallization of glass 2 proved to be very challenging for EBSD-analysis. The triple texture indicated in Fig. 13 as well as the simultaneous oriented nucleation of multiple phases described for both glasses have never been described in the literature concerning glass-ceramics.

In both glasses, nucleation is followed by a very rapid growth selection so that ca. 10 μm below the surface only BTG-crystals with their c-axes perpendicular to the surface prevail while the secondary phases practically disappear. Hence the idea that the degree of orientation is affected by the surface roughness observed after crystallization^9^ may be discredited. Instead the higher intensity detected 100 μm below the surface simply results from the fact that the topmost layer, which shows a different texture, was removed. This orientation selection was also described in ref. 19 via XRD-measurements performed after a stepwise removal of the topmost layer.

As outlined above, it is basically impossible that benitoite and/or BaGe_4_O_9_ play a role similar to that outlined in ref. 21 in the glasses analyzed here. The failure to achieve an adequate polish near the surface similar to that achieved for the analysis of BTS^40^ is probably due to a combination of very small crystal sizes near the surface and high stresses in these samples. The inhomogeneities in the crystal layer described in ref. 17 are very probably a result of these stresses which may cause local fractures during polishing.

The cracks and large number of pores observed in the BTG glass-ceramics, especially at the interface between surface and bulk crystallization in glass 1, increase the difficulty of achieving a high quality surface treatment. The pores at the growth front are very similar to the holes observed during the crystallization of glass fibers with the composition BTG55^22^ where bulk crystallization was not observed, probably because the fibers were only 100–200 μm in diameter. In the glass 1 analyzed here, the oriented layer of growth is frequently more than 500 μm thick, but this thickness probably depends on the glass composition and the temperature/time regime of the crystallization procedure. By contrast, the oriented layer of BTG in glass 2 only reached thicknesses of 30–100 μm in dependence of the annealing temperature.

Due to the basically perfect c-axes orientation observed in the crystallized surface layer featured in the Figs 8 and 9 and 14, it is difficult to imagine how the application of a magnetic field should have a significantly enhancing effect on the degree of orientation after growth selection is complete. As the measurements in ref. 18 were performed on very thin crystal layers less than 10 μm thick, the observed results could indicate that a magnetic field might enhance the velocity of growth selection assuming the results observed here are transferable to other glass compositions in this system.

The uneven growth fronts observed in a number of articles and discussed in the context of nucleation sites^17^ may alternatively be explained by the dendritic growth mechanism observed in the current glass composition. By contrast, the even growth fronts also observed after BTG crystallization in some other glass compositions^13^,^14^,^16^,^18^,^22^ raise the question whether growth in that system occurs via very extremely fine dendrites or rather similar to the growth of STS observed in ref. 39 where dendritic morphologies could not be discerned.

In summary the growth of BTG in both glasses featured here is quite similar to the growth of BTS but widely in contrast to STS in their respective versions of the glass1 composition. The novel observation of a multi-phase oriented nucleation including a triple texture further cement the fact that assuming statistically oriented nucleation in theoretical models concerning crystal nucleation in glasses is questionable. Both BTG and BTS show oriented nucleation with the c-axis tilted by 55 and 58 ±10° respectively for the equivalent compositions. In addition to the temperature dependence of oriented nucleation observed here and in the BTS^41^ system, a composition dependence of the detected textures is indicated for the BTG system. Both phases show dendritic growth and a kinetic selection towards a texture where the c-axis is oriented perpendicular to the surface. A time delayed bulk nucleation is observed in both cases blocking the oriented layer. The thickness of the oriented crystal layers observed in BTG and the size of the homogeneously oriented crystal domains in the bulk indicate that bulk nucleation in BTG is less frequent for glass 1 and exhibits a longer induction time than in the equivalent glass in the BTS system. In contrast to the accelerating growth front interaction observed in STS^32^ colliding growth fronts simply block each other in the analyzed samples. The nucleation order of the phases in the BTG system is probably very sensitive to impurities and/or glass production methods and the secondary phases do not play a significant role during growth into the bulk.

## Methods

Glass 1 with the composition 2 BaO · TiO_2_ · 2.75 GeO_2_ was melted from a mixture of the reagent grade raw materials BaCO_3_, TiO_2_ and GeO_2_ in a Pt-crucible at 1550 °C in an inductive furnace for 2 h and stirred for another 2 h at this temperature to homogenize the melt. The glass was then poured on a brass block, quenched for about 7 s with a brass stamp and subsequently transferred to a furnace preheated to a temperature of 760 °C (which is close to T_g_). The glass 2 was prepared in the same inductive furnace using the same raw materials. It was melted at 1300 °C for 1 h and stirred for another 20 min. After quenching it was transferred to a furnace preheated to 660 °C. The respective furnaces were switched off allowing the glasses to cool with a rate of approximately 3 °C/min. Crystalline powders of BaTiGe_3_O_9_ and BaGe_4_O_9_ were prepared via solid state reactions using BaCO_3_ (VK Labor- und Feinchemikalien, pure), TiO_2_ (Ventron, 99.8%) and GeO_2_ (ABCR GmbH & Co. KG, 99.98%) as raw materials. The stoichiometric mixture of BaTiGe_3_O_9_ was heat treated at 1230 °C while the stoichiometric mixture of BaGe_4_O_9_ was heat treated at 1050 °C where they were held for 20 h with three intermediate regrinding steps.

Cross sections and powders were embedded in Araldite CY212 for further treatment. Samples were manually polished with shrinking grain sizes down to 0.75 μm diamond suspension. A final finish of 30 min using colloidal silica was applied. Conductivity of the surface was achieved by mounting the sample using Ag-paste and applying a thin coating of carbon at about 10^−3^ Pa.

The glass 1 was analyzed by DTA according to Hartmann & Braun. The grain size of the powder was smaller than 70 μm and a heating rate of 10 °C/min was used. Glass 2 was analyzed by DSC using a Linseis DSC Pt 1600 using Pt/Rh10 crucibles and a heating rate of 10 K/min. X-ray diffraction (XRD) was performed using CuKα–radiation in a SIEMENS D5000 diffractometer. SEM analyses were performed using a Jeol JSM 7001F scanning electron microscope equipped with an EDAX Trident analyzing system containing a Digiview 3 EBSD-camera. EBSD-scans were performed using a voltage of 20 kV and a current of ca. 2.40 nA. The scans were captured and evaluated using the software TSL OIM Data Collection 5.31 and TSL OIM Analysis 6.2. All scans were cleaned using the “grain CI standardization” function. Afterwards, unreliable data points were removed by applying a CI filter of 0.1. No further cleanups which actually modify orientations were applied. Pole figures of textures are presented in multiples of a random distribution (MRD).

## Additional Information

**How to cite this article**: Wisniewski, W. *et al.* Oriented Nucleation of both Ge-Fresnoite and Benitoite/BaGe_4_O_9_ during the Surface Crystallisation of Glass Studied by Electron Backscatter Diffraction. *Sci. Rep.* **6**, 20125; doi: 10.1038/srep20125 (2016).

## Figures and Tables

**Table 1:** Glass compositions used to crystallize BTG sorted by GeO_2_ content.

stated composition	Tg [°C]	Tx [°C](bulk)	dielectricconstant	d_33_ [m/V]	References
2 BaO-1 TiO_2_-2 SiO_2_- 1 GeO_2_			11.0	6 × 10^−12^	10
40 BaO-20 TiO_2_-40 GeO_2_	660–669	763		3–7 × 10^−12^	9,17,23
BaO- TiO_2_-GeO_2_			15.0	6–7 × 10^−12^	10,11
40 BaO-15 TiO_2_-45 GeO_2_	658–661	762			23
35 BaO-25 TiO_2_-40 GeO_2_	686–689	791			23
40 BaO-10 TiO_2_-50 GeO_2_	645–646	773			23
35 BaO-20 TiO_2_-45 GeO_2_	677–689	804			23
2 BaO- 1 TiO_2_- 2.75 GeO_2_	673	796			current
35 BaO-15 TiO_2_-50 GeO_2_	660–663	780–783	∼7.0	12 × 10^−12^	15,17,23,24
as above + furher additives	(670)	(780)			28
30 BaO-25 TiO_2_-45 GeO_2_	693–698	814			23
1 Cu-33.3 BaO-16.7 TiO_2_-50 GeO_2_	670	780			29
35 BaO-10 TiO_2_-55 GeO_2_	646–649	775			16,23
30 BaO-20 TiO_2_-50 GeO_2_	676–679	811			23
36 BaO-36 TiO_2_-64 GeO_2_			12.5	2–3 × 10^−12^	8
30 BaO-15 TiO_2_-55 GeO_2_	665–678	809–812	∼10.0	3.7–24.0 ± 3 × 10^−12^	13, 14, 15, 16, 17, 18, 19, 20, 21, 22, 23
as above + furher additives					22
30 BaO-10 TiO_2_-60 GeO_2_	653–654	749			23
25 BaO-20 TiO_2_-55 GeO_2_	680–681	761			23
25 BaO-25 TiO_2_-50 GeO_2_	691–693	813			23
25 BaO-15 TiO_2_-60 GeO_2_	673–676	734			23
20 BaO-20 TiO_2_-60 GeO_2_	669–670	733			23

Selected properties are presented. All values originate from the respective references as only two compositions are analyzed in this article.

**Table 2:** Selected indexing parameters for the best orientations solutions calculated for the EBSD-patterns presented in Fig. 3.

Pattern	Phase	Votes	Fit [°]	CI	comment
1	**BTG**	**113**	**0.86**	**0.475**	const. 001-pole
	benitoite	8	2.18	0.025	
	BaGe_4_O_9_	5	2.33	0.008	
2	**BTG**	**112**	**0.62**	**0.233**	const. 001-pole
	benitoite	6	2.12	0.000	
	BaGe_4_O_9_	4	2.05	0.000	
3	**BTG**	**104**	**0.59**	**0.167**	const. 001-pole
	benitoite	10	1.71	0.042	
	BaGe_4_O_9_	4	2.01	0.000	
4	**BTG**	**84**	**0.68**	**0.233**	const. 001-pole
	benitoite	9	2.22	0.006	
	BaGe_4_O_9_	9	2.14	0.006	
5	BTG	55	1.39	0.212	
	**benitoite**	**56**	**1.04**	**0.279**	
	**BaGe**_**4**_**O**_**9**_	**85**	**1.05**	**0.000**	pseudosymmetry
6	BTG	13	2.14	2.14	
	**benitoite**	**120**	**0.94**	**0.552**	
	**BaGe**_**4**_**O**_**9**_	**120**	**1.21**	**0.000**	pseudosymmetry
7	BTG	32	1.72	0.079	
	**benitoite**	**117**	**0.90**	**0.612**	
	**BaGe**_**4**_**O**_**9**_	**119**	**0.73**	**0.000**	pseudosymmetry
8	BTG	55	1.51	0.036	
	**benitoite**	**120**	**0.70**	**0.521**	
	**BaGe**_**4**_**O**_**9**_	**120**	**0.47**	**0.000**	pseudosymmetry

They were indexed using 10 bands and an IAT of 3 (also used in the scans) in the process. Parameter sets indicating a reliable orientation solution are bold.

**Table 3:** Selected information of the EBSD-datasets used to calculate the textures presented in Fig. 13.

annealing regime	data points	cleaned	in %	%BTG	% Benitoite
3 h @ 690 °C	534188	119886	22.4	3.3	96.7
10 h @ 970 °C	426348	323269	75.8	34.2	65.8
3 h @ 690 °C + 3 h @ 720 °C	605556	43442	7.2	29.4	70.6

## References

[b1] Höche T., Rüssel C. & Neumann W. Incommensurate modulations in Ba_2_TiSi_2_O_8_, Sr_2_TiSi_2_O_8_, and Ba_2_TiGe_2_O_8_ Solid State Commun. 110, 651–656 (1999).

[b2] Schmid H., Genequand P., Tippmann H., Pouilly G. & Guédu H. Pyroelectricity and related properties in the fresnoite pseudobinary system Ba_2_TiGe_2_O_8_-Ba_2_TiSi_2_O_8_ J. Mater. Sci. 13, 2257–2265 (1978).

[b3] Kimura M., Utsumi K. & Nanamatsu S. Ferroelastic behavior in Ba_2_Ge_2_TiO_8_ J. Appl. Phys. 47, 2249–2251 (1976).

[b4] Markgraf S. A. & Bhalla A. S. Low-Temperature Phase Transition in Ba_2_TiGe_2_O_8_ Phase Trans. 18, 55–76 (1989).

[b5] Iijima K., Marumo F., Kimura M. & Kawamura T. Structure of a ferroelastic crystal Ba_2_TiGe_2_O_8_ and its thermal phase transition (in Japanese with English summary) J. Chem. Soc. Jpn. 10, 1557–1563 (1981).

[b6] Höche T., Esmaeilzadeh S., Uecker R., Lidin S. & Neumann W. (3 + 1)-Dimensional structure refinement of the fresnoite framework-structure type compound Ba_2_TiGe_2_O_8_ Acta Cryst. B59, 209–216 (2003). 10.1107/s0108768102021353 12657814

[b7] Halliyal A., Bhalla A. S. & Cross L. E. Phase transitions, dielectric, piezoelectric and pyroelectric properties of barium titanium germanate Ba_2_TiGe_2_O_8_ single crystals Ferroelectrics 62, 3–9 (1985).

[b8] Halliyal A., Bhalla A. S., Newnham R. E. & Cross L. E. Ba_2_TiGe_2_O_8_ and Ba_2_TiSi_2_O_8_ pyroelectric glass-ceramics J. Mater. Sci. 16, 1023–1028 (1981).

[b9] Halliyal A., Bhalla A. S., Newnham R. E., Cross L. E. & Gururaja T. R. Study of the piezoelectric properties of Ba_2_Ge_2_TiO_8_ glass-ceramic and single crystals J. Mater. Sci. 17, 295–300 (1982).

[b10] Halliyal A., Bhalla A. S. & Newnham R. E. Polar Glass Ceramics – A new Family of Electroceramic Materials: Tailoring the Piezoelectric and Pyroelectric Properties Mater. Res. Bull. 18, 1007–1019 (1983).

[b11] Halliyal A., Safari A., Bhalla A. S. & Newnham R. E. Grain-Oriented glass-ceramics: New Materials for hydrophone applications Ferroelectrics 50, 45–50 (1983).

[b12] Halliyal A., Safari A., Bhalla A. S., Newnham R. E. & Cross L. E. Grain-Oriented Glass-Ceramics for Piezoelectric Devices J. Am. Ceram. Soc. 67, 331–335 (1984).

[b13] Takahashi Y., Benino Y., Fujiwara T. & Komatsu T. Large second-order optical nonlinearities of fresnoite-type crystals in transparent surface-crystallized glasses J. Appl. Phys. 95, 3503–3508 (2004).

[b14] Takahashi Y., Benino Y., Fujiwara T. & Komatsu T. Optical second order nonlinearity of transparent Ba_2_TiGe_2_O_8_ crystallized glasses Appl. Phys. Lett. 81, 223–225 (2002).

[b15] Takahashi Y., Benino Y., Fujiwara T. & Komatsu T. Second-Order Optical Nonlinearity of LaBGeO_5_, LiBGeO_4_ and Ba_2_TiGe_2_O_8_ Crystals in Corresponding Crystallized Glasses Jpn. J. Appl. Phys. 41, 1455–1458 (2002).

[b16] Takahashi Y., Benino Y., Fujiwara T. & Komatsu T. Formation mechanism of ferroelastic Ba_2_TiGe_2_O_8_ and second order optical non-linearity in transparent crystallized glasses J. Non-Cryst. Solids 316, 320–330 (2003).

[b17] Takahashi Y., Saitoh K., Benino Y., Fujiwara T. & Komatsu T. Formation of Ba_2_TiGe_2_O_8_ phase in BaO–TiO_2_–GeO_2_ glasses and their optical non-linearities J. Non-Cryst. Solids 245 & 346, 412–416 (2004).

[b18] Toyohara N. *et al.* Enhancement and depression in second-order optical nonlinearity of Ba_2_TiGe_2_O_8_ in crystallized glass prepared in a high magnetic field J. Appl. Phys. 99, 043515 (2006).

[b19] Masai H. & Fujiwara T. Large second-order optical nonlinearity in 30BaO–15TiO_2_–55GeO_2_ surface crystallized glass with strong orientation J. Appl. Phys. 100, 023526 (2006).

[b20] Masai H., Fujiwara T., Benino Y., Komatsu T. & Mori H. Selective surface crystallization of nonstoichiometric 30BaO-15TiO_2_-55GeO_2_ glass J. Appl. Phys. 101, 033530 (2007).

[b21] Masai H., Fujiwara T., Mori H., Benino Y. & Komatsu T. Dual layered surface crystallization of 30BaO–15TiO_2_–55GeO_2_ glass by stepwise heat treatment J. Appl. Phys. 101, 123505 (2007).

[b22] Hane Y., Komatsu T., Benino Y. & Fujiwara T. Transparent nonlinear optical crystallized glass fibers with highly oriented Ba_2_TiGe_2_O_8_ crystals J. Appl. Phys. 103, 063512 (2008).

[b23] Yamazaki Y., Masai H., Takahashi Y. & Fujiwara T. Thermal property of BaO-TiO_2_-GeO_2_ glass and the crystallization behavior Key Eng. Mat. 445, 179–182 (2010).

[b24] Yamazaki Y., Takahashi Y., Ihara R. & Fujiwara T. Surface crystallization of Fresnoite-type crystallized glasses with large thickness J. Ceram. Soc. Japan 119, 757–762 (2011).

[b25] Keding R. & Rüssel C. Electrochemical Nucleation for the Preparation of Oriented Glass Ceramics J. Non-Cryst. Solids 219, 136–141 (1997).

[b26] Keding R. & Rüssel C. The mechanism of electrochemically induced nucleation in glass melts with the composition 2BaO · TiO_2_ · 2.75SiO_2_. J. Non-Cryst. Solids 351, 1441–1446 (2005).

[b27] Wisniewski W., Nagel M., Völksch G. & Rüssel C. New Insights into the Microstructure of Oriented Fresnoite Dendrites in the System Ba_2_TiSi_2_O_8_-SiO_2_ Through Electron Backscatter Diffraction (EBSD) Cryst. Growth. Des. 10, 1939–1945 (2010).

[b28] Wisniewski W., Nagel M. & Rüssel C. Macroscopic Glass-Permeated Single-Crystals of Fresnoite CrystEngComm 17, 2019–2025 (2015).

[b29] Keding R. & Rüssel C. Oriented strontium fresnoite glass-ceramics prepared by electrochemically induced nucleation. J. Mater. Sci. 39, 1433–1435 (2004).

[b30] Honma T., Benino Y., Fujiwara T. & Komatsu T. Transition metal atom heat processing for writing of crystal lines in glass Appl. Phys. Lett. 88, 231105 (2006).

[b31] Honma T., Komatsu T. & Benino Y. Patterning of *c-*axis-oriented Ba_2_TiX_2_O_8_ (*X* = Si, Ge) crystal lines in glass by laser irradiation and their second-order optical nonlinearities J. Mater. Res. 23, 885–887 (2008).

[b32] Imaoka M. & Yamazaki T. Studies of the Glass-formation Range of Germanate Systems J. Ceram. Assoc. Jap. 72, 182–191 (1964).

[b33] Masai H. *et al.* Precipitation of Pt nanocrystallites from BaO-TiO_2_-GeO_2_ remelted glass J. Appl. Phys. 111, 123513 (2012).

[b34] Masai M. *et al.* Effect of melt temperature on the structure of BaO–TiO_2_–GeO_2_ glass Mater. Res. Bull. 47, 4065–4070 (2012).

[b35] Rüssel C. & Freude E. Voltammetric Studies of the Redox Behaviour of Various Multivalent Ions in Soda-Lime-Silica Glass Melts Phys. Chem. Glasses 30, 62–68 (1989).

[b36] Avramov I., Keding R. & Rüssel C. Crystallization Kinetics and Rigidity Percolation in Glass Forming Melts J. Non-Cryst. Solids 272, 147–153 (2000).

[b37] Okamato H. The Ge-Pt System J. Phase Equilib. 13, 413–416 (1992).

[b38] Wisniewski W., Patschger M. & Rüssel C. Sr-fresnoite surface crystallisation in a 2SrO · TiO_2_ · 2.75 SiO_2_ glass studied by EBSD CrystEngComm 14, 5425–5433 (2012).

[b39] Wisniewski W., Takano K., Takahashi Y., Fujiwara T. & Rüssel C. Microstructure of Transparent Strontium Fresnoite Glass-Ceramics Sci. Rep. UK 5, 9069 (2015). 10.1038/srep09069 PMC5376207 25780988

[b40] Wisniewski W., Nagel M., Völksch G. & Rüssel C. Electron Backscatter Diffraction of Fresnoite Crystals Grown from the Surface of a 2BaO · TiO_2_ · 2.75SiO_2_ Glass Cryst. Growth. Des. 10, 1414–1418 (2010).

[b41] Wisniewski W., Bocker C., Kouli M., Nagel M. & Rüssel C. Surface Crystallization of Fresnoite from a Glass Studied by Hot Stage Scanning Electron Microscopy and Electron Backscatter Diffraction Cryst. Growth. Des. 13, 3794–3800 (2013).

[b42] Patschger M., Wisniewski W. & Rüssel C. Piezoelectric glass-ceramics produced via oriented growth of Sr_2_TiSi_2_O_8_ fresnoite: thermal annealing of surface modified quenched glasses CrystEngComm 14, 7368–7373 (2012).

[b43] Wisniewski W., Zscheckel T., Völksch G. & Rüssel C. Electron Backscatter Diffraction of BaAl_2_B_2_O_7_ Crystals Grown from the Surface of a BaO · Al_2_O_3_ · B_2_O_3_ Glass CrystEngComm 12, 3105–3111 (2010).

[b44] Zscheckel T., Wisniewski W., Gebhardt A. & Rüssel C. Mechanisms Counteracting the Growth of Large Grains in Industrial ZnS Grown by Chemical Vapor Deposition *ACS Appl*. Mater. Interfaces 6, 394–400 (2014). 10.1021/am404454r 24283799

[b45] Keshavarzi A., Wisniewski W. & Rüssel C. EBSD and EDX Analyses of a Multiphase Glass-Ceramic Obtained by Crystallizing an Yttrium Aluminosilicate Glass *ACS Appl*. Mater. Interfaces 5, 8531–8536 (2013). 10.1021/am401953j 23848162

[b46] Martin S., Berek S. H., Aneziris C. G., Martin U. & Rafaja D. Pitfalls of local and quantitative phase analysis in partially stabilized zirconia J. Appl. Cryst. 45, 1136–1144 (2012).

[b47] Wisniewski W. & Rüssel C. An Experimental Viewpoint on the Information Depth of EBSD *Scanning* doi: 10.1002/sca.21251 (2015). 26248948

[b48] Wisniewski W., Völksch G. & Rüssel C. The degradation of EBSD-patterns as a tool to investigate surface crystallized glasses and to identify glassy surface layers Ultramicroscopy 11, 1712–1719 (2011). 22088446 10.1016/j.ultramic.2011.09.008

